# Advancements in Hydrogel-Based Drug Sustained Release Systems for Bone Tissue Engineering

**DOI:** 10.3389/fphar.2020.00622

**Published:** 2020-05-06

**Authors:** Yunfan Zhang, Tingting Yu, Liying Peng, Qiannan Sun, Yan Wei, Bing Han

**Affiliations:** ^1^Department of Orthodontics, Peking University School and Hospital of Stomatology & National Engineering Laboratory for Digital and Material Technology of Stomatology & Beijing Key Laboratory of Digital Stomatology, Beijing, China; ^2^Department of Geriatric Dentistry, Peking University School and Hospital of Stomatology, Beijing, China

**Keywords:** hydrogel, sustained drug release, bone tissue engineering, growth factors, mesenchymal stem cells

## Abstract

Bone defects caused by injury, disease, or congenital deformity remain a major health concern, and efficiently regenerating bone is a prominent clinical demand worldwide. However, bone regeneration is an intricate process that requires concerted participation of both cells and bioactive factors. Mimicking physiological bone healing procedures, the sustained release of bioactive molecules plays a vital role in creating an optimal osteogenic microenvironment and achieving promising bone repair outcomes. The utilization of biomaterial scaffolds can positively affect the osteogenesis process by integrating cells with bioactive factors in a proper way. A high water content, tunable physio-mechanical properties, and diverse synthetic strategies make hydrogels ideal cell carriers and controlled drug release reservoirs. Herein, we reviewed the current advancements in hydrogel-based drug sustained release systems that have delivered osteogenesis-inducing peptides, nucleic acids, and other bioactive molecules in bone tissue engineering (BTE).

## Introduction

Bone defects may be caused by various events, including trauma, inflammation, neoplasm resection, congenital deformity, and degeneration (Crane et al., 1995; Spicer et al., 2012). Despite numerous solutions being applied to tackle this issue, clinical demands remain unmet.

To date, autologous bone grafts are still the gold standard and most considered therapeutic strategy for critical-sized bone defects among all restoration methods due to their remarkable osteoconductive and osteoinductive properties. However, *de novo* problems might arise, such as a limited amount of donor tissue, an excessive harvest procedure, and the possibility of postoperative infection of the donor site (Langer and Vacanti, 1993; Betz, 2002; Ahlfeld et al., 2019). Allografts or xenografts usually serve as secondary alternatives, as slower incorporation, immune rejection, and pathogen transmission might occur (Crane et al., 1995; Haugen et al., 2019). Utilizing biocompatible scaffold materials, such as mesenchymal stem cells (MSCs) and/or bioactive factors (Meijer et al., 2007), bone tissue engineering can offer more possibilities. Achieving sufficient and qualified bone formation *via* artificial composites is the grand aim of bone tissue engineering.

Compared with bone harvest operations, MSCs are relatively easy to obtain. These cells exhibit self-renewal, multipotentiality (Prockop, 1997), and immunomodulatory properties (Keating, 2008), which are imperative for bone regeneration. In addition, bioactive factors, for example, cytokines and growth factors (GFs), play a crucial role in new bone formation. Bone morphogenetic proteins (BMPs) are a group of GFs that have been substantially investigated. Recombinant human BMP-2 and BMP-7 is commercially available for limited clinical usage (Nauth et al., 2011). However, naked GFs are vulnerable *in vivo*, and to achieve optimal osteogenic effects, a supraphysiological dose of GFs is required. Paradoxically, diffusion or uncontrolled release of GFs may lead to ectopic bone formation and other complications, including carcinogenicity (Carragee et al., 2011; de Melo Pereira and Habibovic, 2018). Hence, attaining sustained release of bioactive factors is an essential objective for scaffold design to promote the therapeutic efficacy of bone tissue engineering. The scaffold materials not only create a congenial microenvironment to promote MSC biological behaviors but also help to maintain bioactive molecules *in situ*. To date, the controlled release of bioactive factors in bone tissue engineering has been realized by a wide range of biomaterials of different natures and configurations, which provide diverse release profiles in different treatment scenarios (Lee and Shin, 2007).

Hydrogels are a category of highly hydrated 3-dimensional (3D) crosslinked homopolymer, copolymer, or macromer networks that can be cast into different shapes and sizes (Slaughter et al., 2009; Seliktar, 2012). The application of hydrogels in tissue engineering, bone tissue engineering in particular, has been garnering increasing attention. Laden with osteogenic-inducing drugs and sustained release profiles, hydrogels have been suggested to be promising bone tissue engineering biomaterials. In this review, we discuss the progress and limitations of current bone tissue engineering, the advantages of hydrogel-based bone regeneration biomaterials and recent advancements in hydrogel-based drug sustained release systems for bone tissue engineering.

## The Present Challenges of Bone Tissue Engineering

To date, substantial progress has been made in bone regenerative medicine. A variety of biomimetic polymers and inorganic materials with bone-like microarchitecture have been designed with advanced manufacturing methods (Wei et al., 2011; Kim et al., 2017b; Yin et al., 2019), including 3D printing, aiming to achieve superb osteogenic properties as well as accuracy and spatial fitness of critical-sized defects. Light-cured, thermal-setting, pH- or enzyme-sensitive, and other smart biomaterials enable bone tissue engineering to serve in many on-demand circumstances. Varieties of seed cells from different origins including umbilical cord MSCs (UCMSCs), induced pluripotent stem cell-derived MSCs (iPSC-MSCs), and embryonic stem cell-derived MSCs (ESC-MSCs) are successfully applied (Xie et al., 2016; Chen et al., 2018). Multifarious drugs or bioactive factors are delivered *in situ* with different strategies and tailored release profiles, offering osteogenic-friendly environments for relevant cells. Noteworthy, it was reported that MSC-derived exosomes combining scaffolds achieved preferable osteogenesis outcomes (Li et al., 2018), indicating the promising prospect of exosomes-based cell-free bone regeneration.

MSCs from different sources, such as bone marrow and dental tissue, are available for bone tissue engineering. The stem cell niche, 3D microenvironments containing specific biophysical and biochemical signals, maintains the stemness of stem cells *in vivo* (Scadden, 2006; Jones and Wagers, 2008). However, maintaining the viability and stemness of MSCs as well as controlling stem cell fate is a fairly critical issue in regenerative medicine. Substrate-derived stimuli are able to prolong the stemness of stem cells and guide stem cell fate into specific lineages (Fisher et al., 2010; Marklein and Burdick, 2010; Lee et al., 2015). Moreover, as the proliferation and differentiation of MSCs may drive into specific lineages depending on different microenvironmental cues, biochemical stimuli, including cytokines and GFs, are used in a spatiotemporal sequence during the complex and continuous reparative procedure (Samorezov and Alsberg, 2015; Farokhi et al., 2016). Successful bone regeneration requires the proper combination of stimuli that can trigger MSC differentiation and matrix deposition. As the scaffold material itself is capable of combining substrate-derived and biochemical stimuli, biomimetic and bioinspired synthetic materials with sustained drug release systems should be designed to facilitate bone tissue regeneration. Due to the constraints of current knowledge in this field, the research is far from sufficient.

Natural bone fracture healing requires the coordinated participation of osteogenesis and angiogenesis (Collin-Osdoby, 1994; Marsell and Einhorn, 2011). Bioactive factors and signal pathway crosstalk, which mediates the interplay between epithelial cells and osteoprogenitors, has been well summarized (Ramasamy et al., 2016). Likewise, vascularization in bone substitutes is vital for successful bone tissue engineering. Insufficient blood supply may result in undernutrition, hypoxia, and inadequate cell recruitment, leading to the failure of bone tissue engineering. Varieties of assessments and solutions have been summarized (Rouwkema et al., 2008; Das and Botchwey, 2011), yet there is no convincing evidence that the strategies are ample to sustain large tissue constructs, encouraging the proposal of more promising methods.

## The Preponderance of Hydrogels in Bone Tissue Engineering

Ideal bone tissue engineering scaffolds should meet the following criteria: (1) biocompatible, nontoxic and nonimmunogenic; (2) porous-structured; (3) proper mechanical properties, load-bearing ability, and sufficient dimensional stability; and (4) fully degradable, with a degradation rate that matches neotissue formation (Lee and Shin, 2007; Slaughter et al., 2009; Haugen et al., 2019). Numerous inorganic scaffolds, such as metals and bioceramics, have been applied in bone regeneration, yet their lack of cell affinity, unbalanced mechanical properties, and rather poor degradation cannot be ignored (Pearlin et al., 2018).

According to types of raw materials, hydrogels can be briefly categorized into natural and synthetic. It is usually considered that natural hydrogels are more biocompatible and bioactive, while synthetic ones possess more tunable mechanical and degradation properties. 3D-structured, highly water-containing, and biocompatible hydrogels act as excellent extracellular matrix (ECM) analogs. The porous structure of the hydrogel enables substance exchange and cell entrance at the initial stage as well as vascular ingrowth in the follow-up stage. It has been substantially shown that cells are easily suspended within hydrogels, and the viability of the encapsulated cells is highly preserved (Gao et al., 2020; Paez et al., 2020).

MSCs are highly sensitive to physical parameters (Higuchi et al., 2013), including viscoelasticity Engler et al. (2006) and topography (Fiedler et al., 2013), in the surrounding milieus. The stiffness (elastic modulus) of the matrix is believed to contribute greatly to determining stem cell fate. As Engler et al. (2006) demonstrated, 2D-cultured MSCs exhibited osteogenic characteristics when the microenvironmental stiffness was relatively rigid, at 20–40 kPa. However, osteogenesis occurred at 11–30 kPa when MSCs were cultivated 3-dimensionally (Huebsch et al., 2010). Due to flexible synthetic strategies and the range of constituents, hydrogels possess tunable physio-mechanical properties, which could match the desirable ranges of material elasticity, porosity, and degradation rate required for bone tissue engineering (Slaughter et al., 2009). Meanwhile, photodegradable (Lunzer et al., 2018), thermal-sensitive, or pH-sensitive (GhavamiNejad et al., 2016) linkages as well as other advantageous materials could be subtly introduced into hydrogels, which may fabricate a versatile and intelligent composite system to fulfill the growing clinical demands.

On the other hand, bioactive molecules play an important role in bone regenerative medicine. During bone formation, numerous cytokines and GFs are orchestrated in a spatiotemporal manner (Farokhi et al., 2016), which would provide a suitable microenvironment for MSC proliferation and differentiation, as well as recruit progenitors from surrounding tissue and peripheral blood for further restoration. Apart from competent cell carriers, hydrogels can also be employed as promising local drug reservoirs. Multiple schemes have been applied to reach desirable and smart drug delivery kinetics (Lee and Shin, 2007; Slaughter et al., 2009). Non-covalent immobilization strategies are the most commonly used in hydrogel-based drug depots, the drug release rate was mainly determined by parameters such as crosslink density, carrier affinity for drugs, and the matrix degradation profile (Dimatteo et al., 2018). Bioactive factors also could be linked covalently to polymers by which a longer drug retention time would be achieved, and covalent linkages could be broken as reactions of specific external cues, leading an on-demand drug controlled release. Moreover, other sustained release systems like microspheres could be introduced to hydrogel matrix, enabling multiple drug molecules sustained release in sequential or spatiotemporal manners (Chen et al., 2010).

## Hydrogel-Based Drug Sustained Release Systems for Bone Tissue Engineering

Extensive drug and sustained release strategies have been designed for bone tissue engineering. Herein, we introduce studies on hydrogel-based controlled release systems according to the category of bioactive molecule loaded within.

### Peptides

The majority of cytokines, GFs, and hormones that stimulate bone formation are peptides. These biomolecules are produced through the autocrine, paracrine, and endocrine systems, acting concertedly to regulate the complex cascade of bone-related gene expression (Lee and Shin, 2007; Farokhi et al., 2016). Hence, a well-orchestrated sustained release system of these peptides has been pursued in order to present a more biomimetic approach.

#### BMP

With the promoted understanding of the underlying mechanism of osteogenesis (Chen et al., 2012), BMP, as a prominent member of the TGF-β superfamily, has always been a favored candidate for bone tissue engineering applications.

Since some hydrogels are believed to possess inferior osteoconductive properties, Olthof et al. (2018) modified an oligo[poly(ethylene glycol) (PEG) fumarate] (OPF) hydrogel with bisphosphate. BMP-2 was encapsulated in poly(lactic-co-glycolic acid) (PLGA) microspheres. The additional BMP-2 and drug-laden PLGA microspheres were entrapped in the hydrogel matrix. The researchers believed that the anionic groups would produce a strong interaction between the matrix and inorganic phase of the bone as well as enhance BMP-2-induced bone formation. The hydrogel matrix could be functionalized by peptides, which might be beneficial to reduce the dose of encapsulated BMP. In addition, nanofibrous mesh-hydrogel hybrid composites have been applied to reach a proper spatiotemporal release profile (Kolambkar et al., 2011). Shekaran et al. (2014) modified a matrix metalloproteinase (MMP)-degradable peptide crosslinked PEG with an α2β1 integrin-specific peptide (GFOGER). The interaction between integrin and collagen I has been proposed to be vital in osteogenic differentiation and mineralization. It was suggested that the modified matrix is able to support cell adhesion and proliferation and upregulate osteogenic gene expression. Laden with the low dose of BMP-2, robust bone healing was achieved. Along with BMP-2, BMP-7 is considered to be a promising GF in bone formation. An injectable chitosan/β-glycerophosphate (CS/β-GP) hydrogel laden with BMP-7 and antibiotic exhibited preferable reparative effects towards infection-induced bone loss (Zang et al., 2019). Growth differentiation factor-5 (GDF-5), also known as BMP-14, regulates the development of numerous tissue and cell types, including limbs and teeth. Bae et al. (2014) mixed different concentrations of GDF-5 with a light-cured hydrogel matrix. The results showed that GDF-5 improved the osteogenic ability in a dose-dependent manner, as the strongest augmentation was achieved by the hydrogel loaded with the highest concentration.

Apart from adsorption or physical entrapment, electrostatic, hydrophobic, or other interactions have been introduced into the systems to prolong the release of BMPs. Heparin was reported to be a strong binder to BMPs, yet the side effects were not negligible. Heparin mimics, which are usually negatively charged, are supposed to be capable of controlling BMP release. Chondroitin sulfate (Anjum et al., 2016), 2-N,6-O-sulfated chitosan (26SCS)-based nanoparticles (Cao et al., 2014), alginate sulfate (Park et al., 2018) were synthesized by researchers, and satisfactory results were achieved both *in vitro* and *in vivo*. When higher concentrations of heparin mimics were introduced, the release rate of BMP became slower. Seo et al. (2015, 2017) harnessed the ionic and hydrophobic interactions provided by polyphosphazene nanoparticles. They found that release rate of BMPs were controlled by the types and amounts of pendants. Thus, the optimal release profile and osteogenesis outcomes rely on a reasonable proportion of BMP-tethering molecules.

Genetic engineering is another option to obtain long-lasting BMP release. As Lin et al. (2019) described in a manuscript, the *BMP* gene was transduced into human bone marrow-derived stem cells (BMMSCs), obtaining a continuous (up to 56 days) and economical BMP supply. Using visible light-based projection stereolithography (VL-PSL) technology to encapsulate the transduced cells, the researchers were able to fabricate more structurally and geometrically compatible constructs for individualized bone defects, which would be conducive to achieving tissue fusion and bone tissue engineering long-term success.

#### Vascular Epithelial Growth Factor (VEGF)

Vascularization plays a crucial role in both bone development and bone regeneration (Collin-Osdoby, 1994; Olsen et al., 2000). Blood vessels do not solely work as substance exchange pathways; they are also regarded as highly active paracrine organs targeting various progenitors during bone formation and reconstruction (Collin-Osdoby, 1994). VEGF, a key angiogenic growth factor (Carmeliet and Jain, 2011), has been widely used in bone tissue engineering.

The cooperation between VEGF receptors and integrin adhesion receptors has been elucidated in angiogenic regulation. Garcia et al. (2016) engineered a protease-degradable, GFOGER-modified PEG hydrogel as a VEGF depot. They found that covalently linked VEGF remained highly bioactive during a prolonged release period. Whereas it was shown that a GFOGER hydrogel augmented neovascularization regardless of exogenous VEGF, micro computed tomography (micro-CT) showed delivering exogenous VEGF failed to enhance critical-sized bone repair. Heterogenous material composites are manufactured by which we can juggle both timed drug release and osteoconduction. Composed of a 3D multichannel calcium phosphate cement (CPC) and alginate/gellan gum (AlgGG) hydrogel, the CPC/AlgGG biphasic scaffold tethers VEGF *via* the interaction with heparin (Ahlfeld et al., 2019). Despite some remarkable properties observed *in vitro*, significant enhancement by VEGF on new bone formation has not been detected. Amirian et al. (2015) coated VEGF and BMP-2 separately onto gelatin-pectin-biphasic calcium phosphate composites. The results revealed that composites coated with VEGF mainly aided in woven bone formation, and the percent of new bone formation was not greater than those coated with BMP-2.

**Figure 1:**
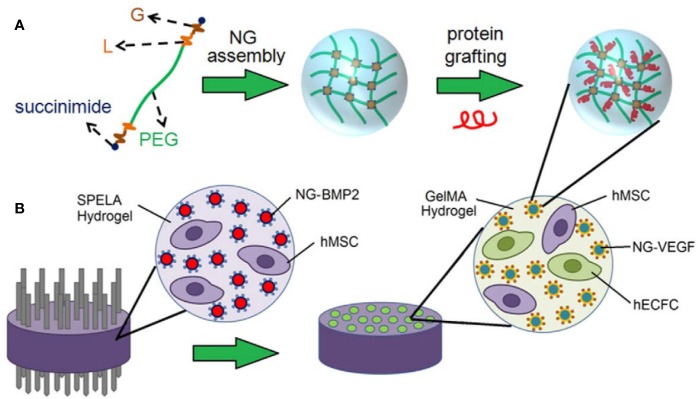
Schematic illustration of **(A)** nanogel (NG) assembly and peptide grafting. **(B)** Achievement of BMP-2 and VEGF spatiotemporal release profiles *via* a patterned hydrogel-based sustained release system. Reprinted from a previous article by Barati et al. (2016) with permission.

Since exclusive delivery of VEGF performed barely satisfactorily in GF-induced osteogenesis, dual or multidrug delivery is warranted. When accompanied by BMP-2, VEGF exhibited a significant improvement in bone formation compared with hydrogels encapsulating BMP-2 alone. VEGF combined with BMP-2 has been used routinely as a GF formula in bone tissue engineering. Similar loading strategies were applied by Barati et al. (2016) and Kader et al. (2019) for spatiotemporal release of BMP-2 and VEGF. MSCs and BMP-tethered nanoparticles were embedded in the outer space, while endothelial colony-forming cells (ECFCs) and VEGF-tethered nanoparticles were dispersed inside the microchannel-patterned hydrogel, as illustrated in **Figure 1**. Degradation and GF release could be tuned by altering stoichiometric ratio chain-extended molecules and proteolysis. According to the data, the release of VEGF and BMP-2 could last over 10 days and 21 days, respectively. It was observed that the patterned hydrogel dual delivery system performed significantly better than that of single delivery systems, which was attributed to paracrine crosstalk. During bone repair, VEGF expression peaks appear in the early period, while BMP peaks later. Thus, consisting of a PLGA microsphere-incorporating poly(propylene fumarate) (PPF) rod surrounded by a rapidly degrading gelatin hydrogel, the composite was designed as a GF delivery vehicle (Kempen et al., 2009). VEGF was encapsulated in the hydrogel, whereas BMP-2 was immobilized by microspheres inside the rod in order to achieve an ideal GF sequential release pattern. VEGF exhibited a large initial burst release within the first 3 days, and BMP-2 showed sustained release over 8 weeks. Likewise, although VEGF did not induce neo-bone formation, it significantly enhanced BMP-induced osteogenesis. Organic-inorganic modular scaffolds are able to optimally orchestrate dual GF release and serve as an “anatomy-structure-function” trinity system in regenerating weight-bearing bones (Bao et al., 2017). Mesoporous bioactive glass (MBG) with hollowed channels and hierarchical porous structures was introduced in a controlled release system as a scaffold (Tang et al., 2020). VEGF was carried by hydrogel inside the channel, and BMP-2 was adsorbed by the MBG scaffolds. 26SCS acted as an analog of ECM, which exhibits super-affinity to GFs. *In vitro* experiments showed that 26SCS promoted the bioactivity of BMP-2 and VEGF. It could be assumed that the VEGF hydrogel column in the hollowed channels might induce chemotaxis of vascular endothelial cells, thus regulating cell migration and vascular infiltration. Moreover, increased type H vessels and neotissue ingrowth were observed.

#### Fibroblast Growth Factor (FGF)

FGF signaling is a dominant regulator during bone development and fracture repair (Bourque et al., 1993; Kronenberg, 2003). However, contradictory results have implied that FGF signaling may exert dual-directional effects on osteogenic procedures, probably in a dose-dependent manner (Kato et al., 1998; Quarto and Longaker, 2006). Thus, sustained release should be achieved when FGF is delivered in bone tissue engineering.

Two Japanese groups encapsulated FGF in gelatin hydrogels for controlled release (Kodama et al., 2009; Furuya et al., 2014). A longer FGF release period may improve cell proliferation, the expression levels of osteogenic markers and BMP-2 as well as bone mineral density (BMD) at defect sites. However, these enhancements vanished, and side effects occurred when a high dose of FGF was delivered (Kodama et al., 2009). In order to achieve bone-like biomechanical properties and slower release of FGF, a stiffer hydrogel matrix, poly(2-hydroxyethyl methacrylate) copolymerized with 2-vinyl pyrrolidone, was engineered (Mabilleau et al., 2008). The data suggested that in the first 4 days, the FGF release rate was approximately 1% per day, which was relevant to hydrogel swelling. Unfortunately, no significant difference between the FGF and control groups was noted in bone mass, but the poorly mineralized woven bone area was significantly larger in the FGF group.

It is a preferable strategy for other GFs to accompany FGF in order to obtain a promising outcome. Chen et al. (2016) chose gelatin microspheres as BMP-2 and basic FGF (bFGF) carriers, which were further embedded in a commercialized injectable thermal-sensitive hydrogel. The hydrogel was injected into a porous cell-loading scaffold before use. Micro-CT revealed that the dual-loaded composites achieved the best reparative results. As expected, composites loading bFGF alone regenerated less bone and neobone at the margin of the defect areas, while the dual-loaded composites showed much more central area bone formation. FGF9 has been indicated to be a stabilizing factor for neovessels, thus, Yuan et al. (2016) introduced FGF9 as an assistant for VEGF, exerting synergetic effects on angiogenesis in bone tissue engineering. A specific peptide segment was fused to VEGF and FGF9 to obtain a covalent connection with the fibrin hydrogel. *BMP-2* was transfected into BMSCs, endowing a greater osteogenic ability and resistance of the osteogenic differentiation inhibition induced by fusion with FGF9. Less bone was formed in the FGF9 groups compared to the groups treated with only VEGF, whereas VEGF/FGF9-loaded composites performed the best among the groups.

#### Other Peptides

Other peptides that regulate the bone regeneration cascade, including osteoprotegerin (OPG) (Jayash et al., 2017), stromal cell-derived factor-1α (SDF-1α) (Ratanavaraporn et al., 2011; Cipitria et al., 2017; Mi et al., 2017), platelet-derived growth factor (PDGF) (Wang et al., 2020), and parathyroid hormone (PTH) (Erten Taysi et al., 2019), etc., might also be worthy of an attempt. The selected studies of hydrogel-based peptide sustained release systems for bone regeneration and their findings are concluded in **Table 1**.

**Table 1:** Summary of selected studies of hydrogel-based peptide sustained release systems.

Peptide	Carrier material	Release pattern	Findings (*ex/in vivo*)	Reference
BMP-2	PLGA microspheres + bisphosphate modified oligo OPF hydrogel	Burst and sustained	Osteoconductivity and osteoinductivity were significantly improved	(Olthof et al., 2018)
BMP-2	Nanofibrous mesh + peptide modified alginate hydrogel	Spatiotemporal controlled release	Micro-CT showed more bone regeneration, superior mechanical properties of neobone were achieved	(Kolambkar et al., 2011)
BMP-2	GFOGER-modified MMP-degradable PEG-maleimide hydrogel	More than 20% BMP-2 remained after 14 days	GFOGER-modified hydrogel exhibited intrinsic osteogenic activities, micro-CT demonstrated; improved bone repair	(Shekaran et al., 2014)
BMP-2	VL-PSL-manufactured live-cell hydrogel scaffold	Sustained expression for 56 days	Micro-CT and histological evidence indicated mature and robust bone formation	(Lin et al., 2019)
BMP-7	CS/β-GP hydrogel	Release 46% in first 12 h, 84% by the end of 336 h	Radiographical and histological observation suggested better periodontal regeneration	(Zang et al., 2019)
GDF-5	Photo-cured hyaluronic acid (HA) hydrogel	Release profiles varies with the initial drug concentration, sustained release period over 25 days	Hydrogel with the highest drug concentration displayed promoted osteogenic potential both *in vitro* and *in vivo*	(Bae et al., 2014)
BMP-2 + VEGF	Acrylate-functionalized lactide-chain-extended star polyethylene glycol (SPELA) hydrogel + gelatin methacryloyl (GelMA) hydrogel + PEG nanogel	Release of VEGF and BMP-2 lasted over 10 days and 21 days, respectively (tunable release kinetics)	Patterned constructs significantly increase osteogenic and vasculogenic differentiation of precursors, bFGF expression was upregulated	(Barati et al., 2016)
VEGF + BMP-2	PLGA microsphere + PPF rod + gelatin hydrogel	A large initial burst was shown *in vivo*, which changed significantly from *ex vivo* release profiles	Micro-CT and histological section demonstrated co-delivery significantly enhanced osteogenesis and angiogenesis ectopically, but it did not reach significant results orthotopically	(Kempen et al., 2009)
VEGF + BMP-2	Hydroxyapatite (HA)/polycaprolactone (PCL) scaffold + PLGA-PEG-PLGA hydrogel	Burst release in first 3 days, sustained release for 3 weeks	Micro-CT showed newly-formed callus in co-delivery group almost covered defect areas, histological analysis showed no significant difference between co-delivery group and autologous group	(Bao et al., 2017)
BMP-2 + VEGF	MBG-based matrix + GelMA/26SCS hydrogel	The release rates of BMP-2 and VEGF were 24.01% and 34.47% respectively within 24 h, 67.90% and 82.73% respectively in 14 days	*In vitro* osteogenic and angiogenic has been markedly improved. Ectopic bone formation in hindlimb ischemia model suggested type H vessels and neobone formation significantly increased	(Tang et al., 2020)
BMP-2 + bFGF	Gelatin microspheres + n-HA/PU40 scaffold + F-127 hydrogel	Pronounced burst release occurred in first 24 h, linear release in following 29 days	Micro-CT analysis indicated dual-delivery reached significantly higher bone volume (BV). Quantitative histological analysis showed remarkable tissue response	(Chen et al., 2016)
VEGF + FGF9	Nanocalcium sulfate + fibrin hydrogel	Addition of the peptide sequence decreased GFs release in an enzyme concentration-dependent manner	Radiographical and quantitative analysis of micro-CT showed the highest BV in dual-delivery hybrid composite. Quantification of blood vessels in explanted tissue suggested more neovessels were obtained	(Yuan et al., 2016)
Osteoprotegerin (OPG)	CS hydrogel	Lasts 28 days, release profile could be adjusted by CS molecular weight	An almost-complete recovery was observed, osteocalcin and osteopontin were upregulated	(Jayash et al., 2017)
SDF-1α	CS/carboxymethyl CS nanoparticles + CS/β-GP hydrogel	20% initial burst release, a cumulative release of 40% over 28 days	Micro-CT showed most new bone formation within the defect area	(Mi et al., 2017)
SDF-1α + BMP-2	Gelatin hydrogel	Large initial burst release of SDF-1α in first 3 days, which may due to BMP-2 combination	Better new bone formation was observed in the dual-delivery group. SDF-1α enhanced BMP-2 osteogenic effects	(Ratanavaraporn et al., 2011)
SDF-1α	RGD-modified alginate hydrogel	Sustained release over 42 days	Improvements induced by SDF-1α or hydrogel stiffness levelled within 8 weeks. Higher number of cells were recruited by SDF-1α, but the difference was not significant *in vivo*	(Cipitria et al., 2017)
PDGF-BB +BMP-9	Sericin hydrogel (genetically incorporated)	Almost 48% released within 17 days, intermittent rapid and slow release phases	Biocompatible compared with other materials and stimulated cell proliferation. Osteogenic markers were significantly upregulated, and greater bone formation when accompanied by BMP-9.	(Wang et al., 2020)
PTH	CS microsphere suspended in poloxamer hydrogel	43% of PTH released in first week, sustained release lasted over 27 days	New bone formation was found to be significantly higher compared to other groups after 10 days, but on day 21 a significant difference exists only when compared with the no treatment group	(Erten Taysi et al., 2019)
Abaloparatide (analog of PTH)	Photo-crosslinked methacrylated gelatin hydrogel	25% released within 24 h, remaining was released steadily over next 10 days	Drug-loaded hydrogel showed significantly higher rate of bone regeneration	(Ning et al., 2019)
Oxytocin	PLGA microsphere + poloxamer hydrogel + β‐tricalcium phosphate (β-TCP) and hydroxyapatite	42% released in first week, complete release within 32 days	4 weeks after operation, the lowest residual graft and highest BMD and BV was obtained among all groups	(Akay et al., 2020)
Calcium accumulating peptide (artificially synthesized)	Gelatin-derived hydrogel	Sustained release over 7 days, collagenase accelerated release	Bone formation markers expression levels were enhanced. Micro-CT and histology showed the regenerative effect was superior to that of BMP-2 hydrogels	(Jo et al., 2018)

### Nucleic Acids

Since GFs and cytokines are required for weeks during new bone formation, gene therapy might be a feasible alternative. Delivering DNA or RNA locally to increase or knockdown target gene expression, gene therapy is capable of manipulating the microenvironment and determining cell fate in bone regenerative medicine.

Fang et al. (1996) utilized collagen sponges as BMP-4 and PTH plasmid DNA carriers to regenerate nonunion rat femur defects early in 1996. Bonadio et al. (1999) confirmed that non-viral DNA delivery possesses numerous advantages compared with the protein strategy. Hydrophilic nucleic acids and hydrogels could provide stable and sequestered environments for gene delivery. Komatsu et al. (2016) demonstrated that gelatin hydrogels could transduce BMP-2 plasmid DNA efficiently, facilitating local bone regeneration. CS or polyethyleneimine (PEI) is usually introduced as the carrier due to the electrostatic interaction between the negatively charged nucleic acids and the polycations. It was reported that branched PEI-HA-DNA complexes were entrapped in bilayered OPF hydrogels to restore osteochondral defects (Needham et al., 2014). Moreover, BMP-2 plasmid DNA conjugated with CS nanoparticles exhibited significant augmentation in hydrogel-mediated rat calvaria bone regeneration (Li et al., 2017). Due to the low stability of liposomes and electrostatic disturbance of other charged compounds, calcium phosphate (CaP) can also be used for DNA incorporation and transfection in bone tissue engineering (Krebs et al., 2010).

MicroRNAs (miRNAs) and small interfering RNAs (siRNAs) are groups of short single-stranded RNA fragments that downregulate target gene expression post-translationally. Various miRNAs associated with bone formation have been reported (Fang et al., 2015), shedding new light on future bone tissue engineering. Nguyen et al. (2014) synthesized an 8-arm PEG *in situ*-forming hydrogel loaded with siRNA/PEI nanocomplexes. siRNA remained bioactive during the prolonged release period. The *in vitro* results showed that siNoggin and siNoggin/miRNA-20a sustained release promoted hMSC osteogenic differentiation in 3D hydrogel cultivation. As mentioned previously, a stiffer substrate may lead to MSC osteogenic differentiation. Carthew et al. (2020) incorporated PEG/gelatin norbornene hydrogels with mechanosensitive miRNAs. MSCs encapsulated in hydrogels were transfected *in situ*, which predominantly enhanced osteogenic gene expression and mineralization. Researchers presumed that the higher transfection efficacy might be ascribed to longer cell exposure times to the transfection agent.

### Ions or Small Molecules

To date, a number of metal ions and artificially synthesized compounds have been found to be beneficial in bone regeneration. Achieving a sustained release pattern and longer duration of drug function may lead to promising therapeutic outcomes.

#### Metal Ions

**Figure 2:**
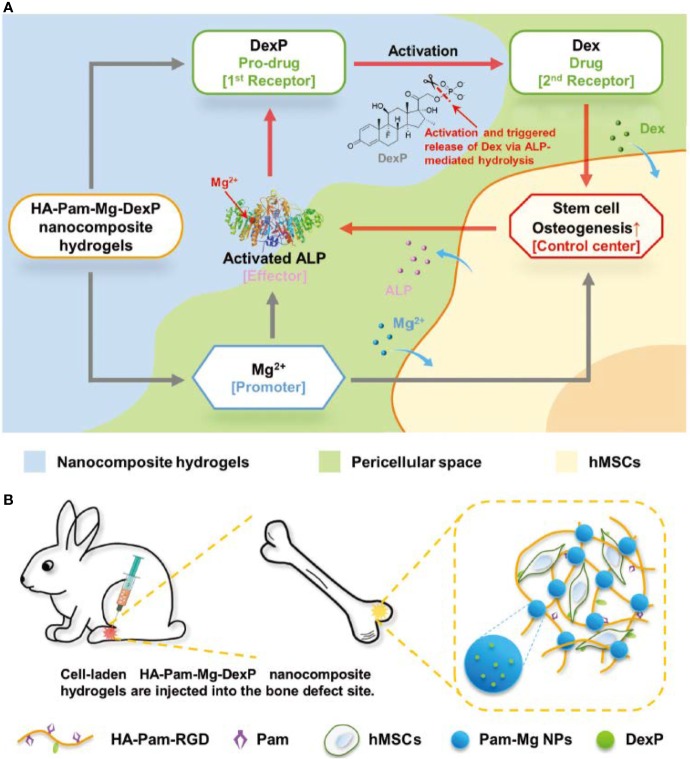
Schematic illustration of **(A)** positive feedback mediated by a cofactor-assisted smart hydrogel drug release system and **(B)** *in situ* application to promote bone regeneration. Reprinted from a previous article by Zhang et al. (2018) with permission.

Since magnesium ions (Mg^2+^) play an important role in bone metabolism and mineralization, a variety of strategies for the sustained delivery of Mg^2+^ have been applied to hydrogel-based scaffolds. Lin et al. (2018b) coated MgO nanoparticles with PLGA and an alginate hydrogel, constructing a monodisperse core-shell delivery system. The release profile of Mg^2+^ revealed a significant suppression of the initial burst, and its release rate was stable and programmed. Enhancement of progenitor cell viability and proliferation, upregulation of osteogenic gene expression levels, and increased bone regeneration volume *in vivo* were attributed to the stable and precise Mg^2+^ supply. Bisphosphonates (BPs) possess two adjacent phosphonic groups, which are propitiously bind to divalent metal ions. Zhang and colleagues (Zhang et al., 2017) developed acrylated-BP-Mg nanoparticles to deliver Mg^2+^ as well as strengthen the acellular hydrogel composite, which serves as a matrix for *in situ* bone formation, *via* multivalent crosslinked domains. They also utilized Mg^2+^ to fulfill on-demand intelligent drug release in bone tissue engineering (Zhang et al., 2018). Intriguingly, Mg^2+^ played multiple roles in this research. First, BP-Mg nanoparticles enabled hydrogel formation and stabilized the prodrug. Second, Mg^2+^ promoted osteogenic differentiation, resulting in increased alkaline phosphatase (ALP) expression. However, and more importantly, Mg^2+^ is also a critical cofactor of ALP. ALP enzymatic hydrolysis was promoted; thus, more bioactive drug molecules were generated, which introduced positive feedback (**Figure 2**). According to the results, this strategy significantly enhanced bone regeneration.

Other metal ions, such as strontium ions (Sr^2+^) and cobalt ions (Co^2+^), may act synergistically in bone reconstruction. A Sr^2+^-crosslinked RGD-alginate hydrogel combined with Sr-doped hydroxyapatite microspheres was engineered, showing a sustained release of Sr^2+^ from two sources (Lourenco et al., 2019). The researchers elaborated that this Sr-hybrid system facilitated MSC osteogenic differentiation, inhibited the functions of osteoclasts and modulated the inflammatory response. As a pro-vasculogenic factor, Co^2+^ was incorporated into the alginate hydrogel shell, while BMP-2 was laden into the collagen core (Perez et al., 2015). Co^2+^ released relatively rapidly, as expected. VEGF secretion and qPCR revealed that Co^2+^ not only stimulated angiogenesis but also elevated osteogenic gene expression. These results indicated an appealing prospect for applying metal ions bone tissue engineering in the future.

#### Small Molecules

A range of pharmaceutical molecules were designed or discovered to be effective in bone regeneration. Highlighted as chelating agents, BPs are utilized as antiresorptive drugs frequently in clinics. BPs mainly target osteoclasts, impeding the differentiation and maturation of osteoclast progenitors. Increasing evidence has shown that BPs directly or indirectly take part in other bone-forming mechanisms and are capable of targeting various cells (Corrado et al., 2017). Since bone healing and regeneration is known to consist of three consecutive phases of inflammation, repair, and remodeling, a proper scale of immune response is indispensable (Claes et al., 2012). However, excess or aberrant immune activation may jeopardize bone repair procedures (Claes et al., 2012; Gibon et al., 2017). Therefore, immunomodulatory drugs, such as nonsteroidal anti-inflammatory drugs (NSAIDs), have been applied in bone tissue engineering. Evidence has shown that aspirin elevates MSC osteogenic potency by inhibiting the tumor necrosis factor-α (TNF-α) and interferon-γ (IFN-γ) pathways (Liu et al., 2011). Statins are inhibitors of a key enzyme of cholesterol synthesis and are widely used to lower serum lipids. Researchers have reported that osteogenesis was enhanced concomitant with promoted *BMP-2* expression in bone cells when treating cells and rodents with statins (Mundy et al., 1999). Localized and sustained delivery of these drugs *via* hydrogels has pointed to a new direction in bone tissue engineering. Many relevant studies are listed and outlined in **Table 2**.

**Table 2:** Summary of selected studies on hydrogel-based small bioactive compound sustained release systems.

Drug	Carrier material	Release pattern	Findings (***ex/in vivo***)	Reference
Alendronate	Fibrin hydrogel	Steady release rate, cumulative release of approximately 45% over 10 days	Hydrogel containing 10^-6^ M showed the best augmentation in cell proliferation, osteogenic differentiation, and bone regeneration.	(Kim et al., 2017a)
Dexamethasone (Dex)	DNA- 2D silicate nanodisks (nSi) hybrid hydrogel	Release rate decreased with higher nSi concentration. Half-time of release was measured from 2.5 to 5.5 days	Drug bioactivity was preserved by the hydrogel. nSi may contribute to *in vivo* osteogenesis whereas Dex showed limited effects.	(Basu et al., 2018)
Aspirin	Thermo-sensitive alginate/β-TCP hydrogel composite	20% drug released in the first day, 40% in 3 days, slowdown in day 5.	Percent of mineralized tissue was significantly higher compared to control group.	(Fang et al., 2018)
Aspirin	Tetra-PEG hydrogel	Released approximately 40% in first 2 days, cumulative release of 80% in 14 days	Low cytotoxicity, significantly improved expression of osteogenic markers and calvarial defect regeneration. Relatively low local inflammation status might be attributed to being laden with aspirin.	(Zhang et al., 2019)
Diclofenac	CS-coated alginate hydrogel	Released 50% and 90% in 2.5 h and 8 h, respectively	Osteoblasts grew and mineralized significantly regardless of drug exhaustion. Osteogenic genes increased over time, while osteogenic suppressing gene expression decreased.	(Lin et al., 2018a)
Tacrolimus	Type I collagen hydrogel	21 days release profile remained similar for different concentrations. Steady release rate.	More newly-formed bone and blood vessels were observed	(Nabavi et al., 2020)
Simvastatin	Maltodextrin micelle-CHO/hydrogel composite	Slow release profile, exhibiting a slight difference according to different degrees of oxidation	Good biocompatibility, stimulated ALP activity and mineralization	(Yan et al., 2018)
Simvastatin	L-lactic acid oligomer (LAo) modified gelatin micelle/gelatin hydrogel composite	Drug released faster as hydrogel crosslinking degree decreased. Release rate showed a good correlation with hydrogel degradation rate.	Hydrogel loaded with 10 μg of drug formed the largest area of bone	(Tanigo et al., 2010)
Rosuvastatin	chitosan/chondroitin sulfate nanoparticles+ Pluronic F127/hyaluronic acid hydrogel composite	Release rate significantly slower than control groups. 60% released from composite in 48 h	Low cytotoxicity, more calcium deposits were observed	(Rezazadeh et al., 2019)

## Conclusion and Future Perspectives

In this review, we summarized a series of investigations focused on hydrogel-based drug sustained release systems in bone tissue engineering. The hydrogels possess a porous microarchitecture, tunable biophysical parameters, and an adjustable degradation rate, which makes them qualified bone tissue engineering scaffolds. Due to their high water content, chemical inertness and relatively sequestered and stable internal environment, they are also excellent in preserving the viabilities of the laden cells and bioactive factors. With the combination of biophysical and biochemical cues, researchers are able to facilely establish an osteo-friendly microenvironment, which would be beneficial for osteoprogenitors to obtain better bone regeneration. Thus, hydrogel-based biomaterials are strong candidates for current or future bone tissue engineering.

Evidence has shown that hydrogel-based drug sustained release systems are highly biocompatible and versatile drug deliverers, obtaining satisfactory osteogenesis results both *in vitro* and *in vivo*. The drug release profile varies according to the loading strategy, degradation ability of the matrix and drug concentration. Among these studies, physical entrapment and diffusion are the most applied drug loading and release strategies, respectively. In particular, the dispersion of drugs, ions and small molecules largely depends on hydrogel pore size and crosslinking density. Although it is quite simple and easy to operate, there are difficulties in initial burst release management. Swelling or degradation of hydrogel matrices contributes to polymer mesh size enlargement, resulting in drug release acceleration, especially for macromolecular drugs. Stronger interactions between matrices and drugs, such as electrostatic interactions and covalent bonds, and other drug reservoirs could be introduced into hydrogels, providing more efficacious drug protection and immobilization. However, negative results have been reported from sustained release systems that did not facilitate bone formation mainly because the carrier exhibited an extremely strong affinity towards the growth factor, resulting in a low level of drug concentration in the surrounding tissue (Hettiaratchi et al., 2017). Thus, optimal drug concentrations should also be determined to achieve a more reasonable and effective release profile.

As mentioned above, cells from different origins are involved in the bone formation process. A vast number of GFs and cytokines collaboratively trigger the repair cascade. Extensive studies have already been conducted on multiple bioactive factors controlled release. Spatiotemporal sequence release of bioactive factors might be a better mimic of complex regeneration procedures as well as exert extraordinary synergistic effects on bone regeneration. Various of multiple GFs delivery strategies was coherently summarized (Chen et al., 2010). Nevertheless, controlling dose ratio of drugs to maximize the synergistic effects and manipulating multiple bioactive factors release kinetics to mimic physiological release profile in different phases of bone regeneration are obstacles in nowadays bone tissue regeneration which needs further investigation.

## Author Contributions

YZ and TY contributed equally to this review. TY, YW, and BH designed and revised this article. YZ, TY, LP, and QS collected the literatures, arranged the outline of collected documents, and wrote the articles. All authors reviewed and commented on the entire manuscript.

## Funding

This work was supported by the National Natural Science Foundation of China (51972005, 51672009, 51903003), National Natural Science Foundation of China Youth Fund (81922019), and National Youth Top-notch Talent Support Program (QNBJ2019-3).

## Conflict of Interest

The authors declare that the research was conducted in the absence of any commercial or financial relationships that could be construed as a potential conflict of interest.
